# Integrated bioinformatics analysis uncovers characteristic genes and molecular subtyping system for endometriosis

**DOI:** 10.3389/fphar.2022.932526

**Published:** 2022-08-17

**Authors:** Zhaowei Wang, Jia Liu, Miaoli Li, Lishan Lian, Xiaojie Cui, Tai-Wei Ng, Maoshu Zhu

**Affiliations:** The Fifth Hospital of Xiamen, Xiamen, Fujian, China

**Keywords:** endometriosis, characteristic genes, molecular subtypes, diagnosis, immune characteristics

## Abstract

**Objective:** Endometriosis is a chronic inflammatory estrogen-dependent disease with the growth of endometrial tissues outside the uterine cavity. Nevertheless, the etiology of endometriosis is still unclear. Integrated bioinformatics analysis was implemented to reveal the molecular mechanisms underlying this disease.

**Methods:** A total of four gene expression datasets (GSE7305, GSE11691, GSE23339, and GSE25628) were retrieved from the GEO, which were merged into a meta-dataset, followed by the removal of batch effects via the sva package. Weighted gene co-expression network analysis (WGCNA) was implemented, and endometriosis-related genes were screened under normal and endometriosis conditions. Thereafter, characteristic genes were determined via Lasso analysis. The diagnostic performance was estimated via receiver operating characteristic curves, and epigenetic and post-transcriptional modifications were analyzed. Small molecular compounds were predicted. Unsupervised clustering analysis was conducted via non-negative matrix factorization algorithm. The enriched pathways were analyzed via gene set enrichment analysis or GSVA. Immune features were evaluated according to immune-checkpoints, HLA, receptors, chemokines, and immune cells.

**Results:** In total, four characteristic genes (BGN, AQP1, ELMO1, and DDR2) were determined for endometriosis, all of which exhibited the favorable efficacy in diagnosing endometriosis. Their aberrant levels were modulated by epigenetic and post-transcriptional modifications. In total, 51 potential drugs were predicted against endometriosis. The characteristic genes exhibited remarkable associations with immunological function. Three subtypes were classified across endometriosis, with different mechanisms and immune features.

**Conclusion:** Our study reveals the characteristic genes and novel molecular subtyping of endometriosis, contributing to the early diagnosis and intervention in endometriosis.

## Introduction

Endometriosis is a chronic inflammatory estrogen-dependent disease caused by functional endometrial tissue that grows outside the uterine cavity (Hung et al., 2021). Typical symptoms involve chronic pelvic pain and abnormal menstruation as well as dyspareunia (Bunis et al., 2021). Endometriosis is frequent among women of childbearing age, with an incidence of about 10% (Symons et al., 2018). About 40–60% of endometriosis cases have dysmenorrhea, while 20–30% have infertility (Bedaiwy, 2022). The present therapies of endometriosis comprise surgery and medicines. Conservative surgery not only enables to remove endometriotic deposits but also enhances the risks of compromising ovarian reserve, which harms other organs as well as imposes postoperative relapse (Hey-Cunningham et al., 2022). Medicines that contain hormonal or nonhormonal therapies depend upon distinct factors (severity of symptoms, willingness to conceive, and comorbidities, etc.) (Brichant et al., 2021). Currently, no drugs are capable of curing endometriosis, and symptoms recur once the drug is discontinued. As a consequence, it is crucial to uncover the aberrant molecular pathways during endometriosis progression as well as determine and develop novel pharmaceuticals for endometriosis.

Endometriotic lesions contain an extremely complex and dynamic environment dominated by inflammation, angiogenesis, and endocrine signaling (Hirakawa et al., 2016). A variety of pathogenic mechanisms result in endometriosis initiation, with much research exploring the reason behind its progression, containing physical factors (uterine tissue injury or scars, residual cell populations in menstrual blood, stem cell populations, and uterine environment, etc.) as well as biochemical factors (angiogenesis, etc.) (Kapoor et al., 2021). It is of importance to probe the key mechanisms responsible for endometriosis. Through illustrating the molecular mechanisms underlying endometriosis, it is of possibility to determine the future candidate pathways for endometriosis therapies. Our study determined characteristic genes of endometriosis via integration of weighted gene co-expression network analysis (WGCNA) and Lasso approaches, as well as classified endometriosis into three distinct subtypes via a non-negative matrix factorization (NMF) clustering approach, assisting to comprehend the mechanisms underlying endometriosis.

## Materials and methods

### Endometriosis datasets and preprocessing

Human endometriosis gene expression datasets were retrieved from the Gene Expression Omnibus (GEO; https://www.ncbi.nlm.nih.gov/gds/). In total, four available datasets (GSE7305 (10 normal endometrium tissues and 10 diseased endometrium tissues) (Hever et al., 2007), GSE11691 (9 normal endometrium tissues and 9 diseased endometrium tissues) (Hull et al., 2008), GSE23339 (9 normal endometrium tissues and 10 diseased endometrium tissues) (Hawkins et al., 2011), and GSE25628 (6 normal endometrium tissues and 16 diseased endometrium tissues) (Crispi et al., 2013)) were collected. The raw “CEL” files of aforementioned datasets were downloaded, which were adjusted for the background and normalized with affy (Gautier et al., 2004) and simpleaffy (Wilson and Miller, 2005) packages. Thereafter, these datasets were merged into a meta-dataset, and then the batch effects were removed via the sva package (Supplementary Figures S1A,B) (Leek et al., 2012). Additionally, the GSE7846 dataset comprising expression profiling of endometrial endothelial cells from five endometriosis patients and five controls was utilized as an external verification set.

### WGCNA

The WGCNA package (Langfelder and Horvath, 2008) was employed for constructing the co-expression networks as well as determining the endometriosis-related modules. Hierarchical clustering analysis was implemented, followed by the removal of outlier specimens. The appropriate soft-thresholding power was computed, and the scale-free networks were built. The co-expression modules were clustered with a dynamic tree-cut approach. The endometriosis-related genes in the modules that were highly correlated to endometriosis were determined. Thereafter, correlation analysis of module membership with gene significance was implemented.

### Functional and pathway enrichment analysis

Functional annotation of endometriosis-related genes was implemented via the clusterProfiler package (Yu et al., 2012). *p* < 0.05 indicated significant enrichment of Gene Ontology and KEGG. Through the GSVA package (Hänzelmann et al., 2013), the enrichment analysis was conducted for ascertaining the difference in pathways among distinct clusters. The gene sets of “c2. cp.kegg.v7.5.1. symbols” and “c5. go.bp.v7.5.1. symbols” were acquired from the Molecular Signatures Database to run GSVA enrichment analysis (Liberzon et al., 2015).

### Screening characteristic genes

Through Lasso Cox regression algorithm, over-fitting risk was minimized with the glmnet package. The alteration trajectory of each variable was assessed and 10-fold cross-validated. Thereafter, characteristic genes were determined, which were subjected to the generation of receiver operating characteristic (ROC) curves.

### Construction of a nomogram

A predictive nomogram was constructed with the rms package. In the nomogram, each variable corresponded to a score, and the total score was computed through adding the scores for all variables (Chen et al., 2021). A calibration diagram of the nomogram was implemented for depicting the diagnostic value of the nomogram-predicted and virtually observed outcome.

### Gene set enrichment analysis

To analyze the biological pathways enriched in high or low level of each characteristic gene, GSEA software was employed with default parameters (Subramanian et al., 2005). The cutoff point of each gene was determined as the median expression value. The most enriched pathways were visualized.

### Analysis of epigenetic and post-transcriptional modifications

Associations of DNA methylation and m^6^A regulators with characteristic genes were evaluated with Pearson correlation tests. MiRNAs with differential expression between normal and diseased endometrium tissues were screened with the false discovery rate (FDR) < 0.05. Thereafter, targeted mRNAs of these miRNAs were then predicted, which were intersected with characteristic genes.

### Prediction of potential drugs

Genes with differential expression between normal and diseased endometrium tissues were determined in accordance with |log fold-change| >1 and FDR <0.05 via the limma package (Ritchie et al., 2015). The up- or downregulated genes were uploaded onto the Connectivity Map (Cmap) database (Yang et al., 2013). Scores that ranged from −1 to 1 demonstrated the correlations of compounds with the aforementioned genes. Compounds with scores ≤ −0.75 were considered potential drugs against endometriosis.

### Evaluation of immune features

The gene sets of immune-checkpoints, HLA, receptors, and chemokines were collected. Through running CIBERSORT algorithm, the relative proportions of 22 immune compositions were estimated (Newman et al., 2015). On the basis of a gene expression matrix as well as specific gene sets of 22 immune cell compositions, the simulation calculation was implemented 1,000 times. The relative composition ratios of these immune cells across each tissue were computed. Immune and stromal scores of each tissue were computed with the ESTIMATE algorithm (Yoshihara et al., 2013).

### Non-negative matrix factorization clustering analysis

Endometriosis-related genes were utilized for NMF clustering analysis, and clusters were determined in the meta-cohort. The k-value where the magnitude of the cophenetic correlation coefficients began to fall was determined as the optimal number of clusters. The heatmaps of endometriosis-related genes and basis component as well as connectivity matrix of NMF in each cluster were evaluated via the NMF package (Pan and Gillis, 2021). Principal component analysis (PCA) was depicted with the ggplot2 package.

### Statistical analysis

Statistical analysis was implemented with R version 4.1.0, with two-sided *p*-value ≤0.05. Student’s t, Wilcoxon, Kruskal–Wallis, or one-way ANOVA test was utilized for estimating the differences of variables between groups. The area under the curve (AUC) values were computed for estimating the predictive power of each characteristic gene. The Spearman or Pearson correlation test was conducted to estimate the relationships between variables.

## Results

### Co-expression analysis of endometriosis and normal endometrium tissues

**FIGURE 1:**
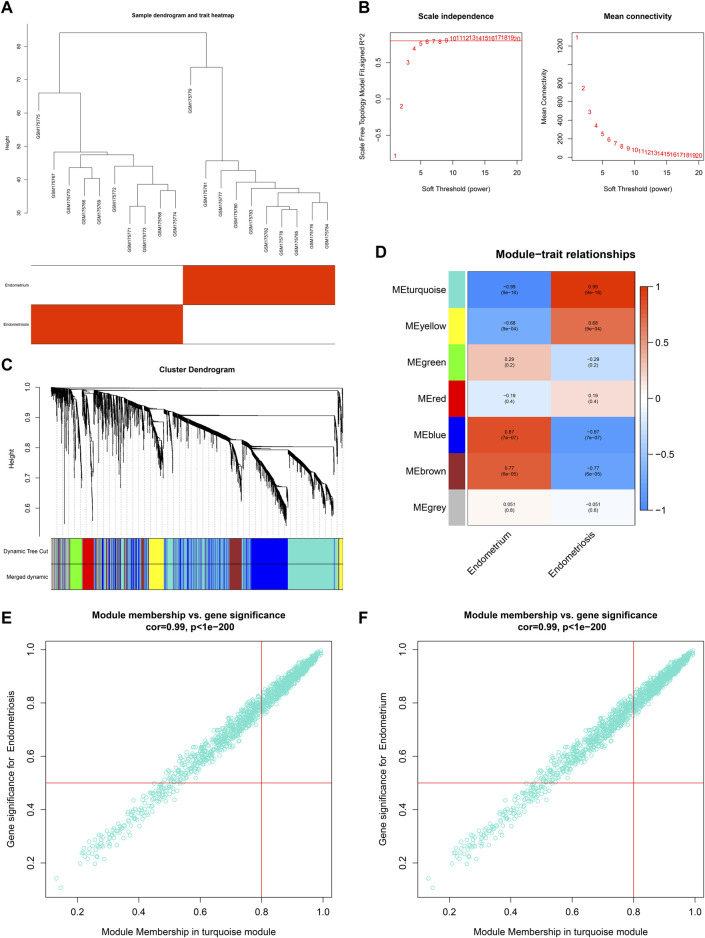
Co-expression analysis in the GSE7305 dataset. **(A)** Clustering dendrograms of specimens. **(B)** Determining the weighted value *β* that satisfied a scale-free network. **(C)** Co-expression module clustering. Each branch represented each gene, and genes clustered into the same module were assigned the same color. **(D)** Correlations of modules with normal endometrium and endometriosis conditions. **(E,F)** Scatter plots for the relationships of module membership in the turquoise module with gene significance for **(E)** endometriosis or **(F)** normal endometrium.

Co-expression analysis was implemented in two public datasets: GSE7305 and GSE11691. For the GSE7305 dataset, we first conducted clustering dendrograms of 10 normal endometrium tissues and 10 diseased endometrium tissues, with no outliers (Figure 1A). The weighted value *β* satisfied a scale-free network (Figure 1B). The seven co-expression modules were merged (Figure 1C), containing blue module (847 genes), brown module (273 genes), red module (129 genes), green module (156 genes), yellow module (231 genes), turquoise module (1,283 genes), and gray module (93 genes). Among them, the turquoise module had the strongest positive association with endometriosis (r = 0.99, p = 9e-18) as well as the strongest negative association with normal endometrium (r = -0.99, p = 9e-18) (Figure 1D). Additionally, the module membership in the turquoise module was strongly linked to gene significance for endometriosis or normal endometrium (Figures 1E,F). Hence, the genes in the turquoise module were identified as endometriosis-related genes in the GSE7305 dataset.

**FIGURE 2:**
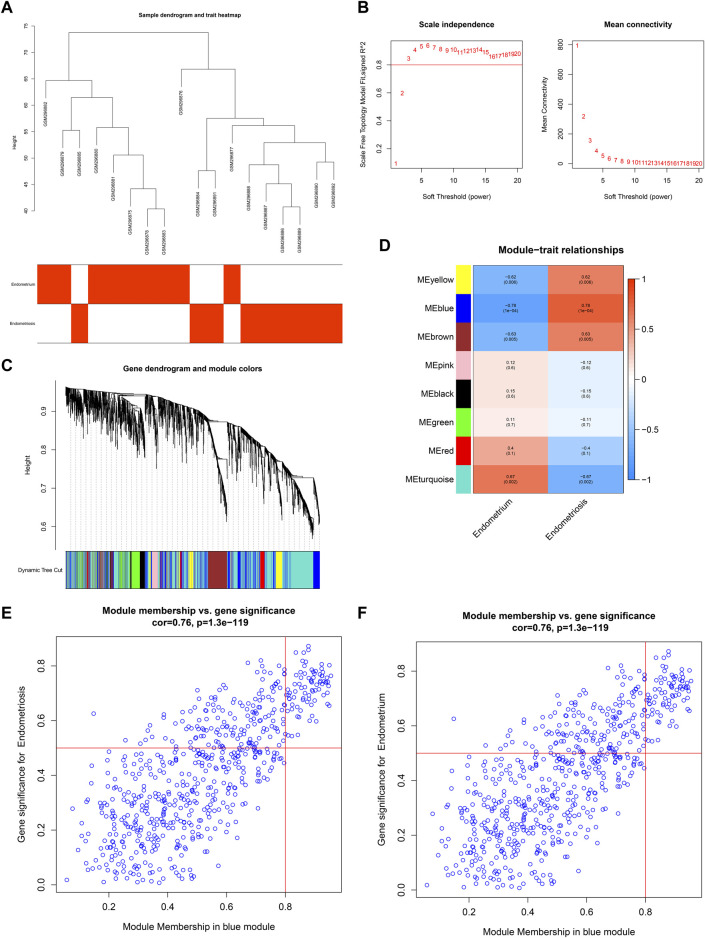
Co-expression analysis in the GSE11691 dataset. **(A)** Clustering dendrograms of samples. **(B)** Identifying *β*-value that satisfied a scale-free network. **(C)** Co-expression module clustering. **(D)** Correlations of modules with normal endometrium and endometriosis conditions. **(E,F)** Scatter plots for the relationships of module membership in the blue module with gene significance for **(E)** endometriosis or **(F)** normal endometrium.

For the GSE11691 dataset, no outliers were detected among nine normal endometrium tissues and nine diseased endometrium tissues (Figure 2A). The *β*-value was set at 3, which satisfied a scale-free network (Figure 2B). Eight co-expression modules were identified (Figure 2C), turquoise module (1,315 genes), red module (111 genes), green module (229 genes), black module (110 genes), pink module (74 genes), brown module (298 genes), blue module (632 genes), and yellow module (251 genes). Among them, the blue module displayed the strongest positive correlation with endometriosis (*r* = 0.78, *p* = 1e-04) as well as the strongest negative correlation with normal endometrium (*r* = −0.78, *p* = 1e-04) (Figure 2D). As depicted in Figures 2E,F, the module membership in the blue module was strongly associated with gene significance for endometriosis or normal endometrium. Thus, the genes in the blue module were identified as endometriosis-related genes in the GSE11691 dataset.

### Identification of shared endometriosis-related genes in two datasets

**FIGURE 3:**
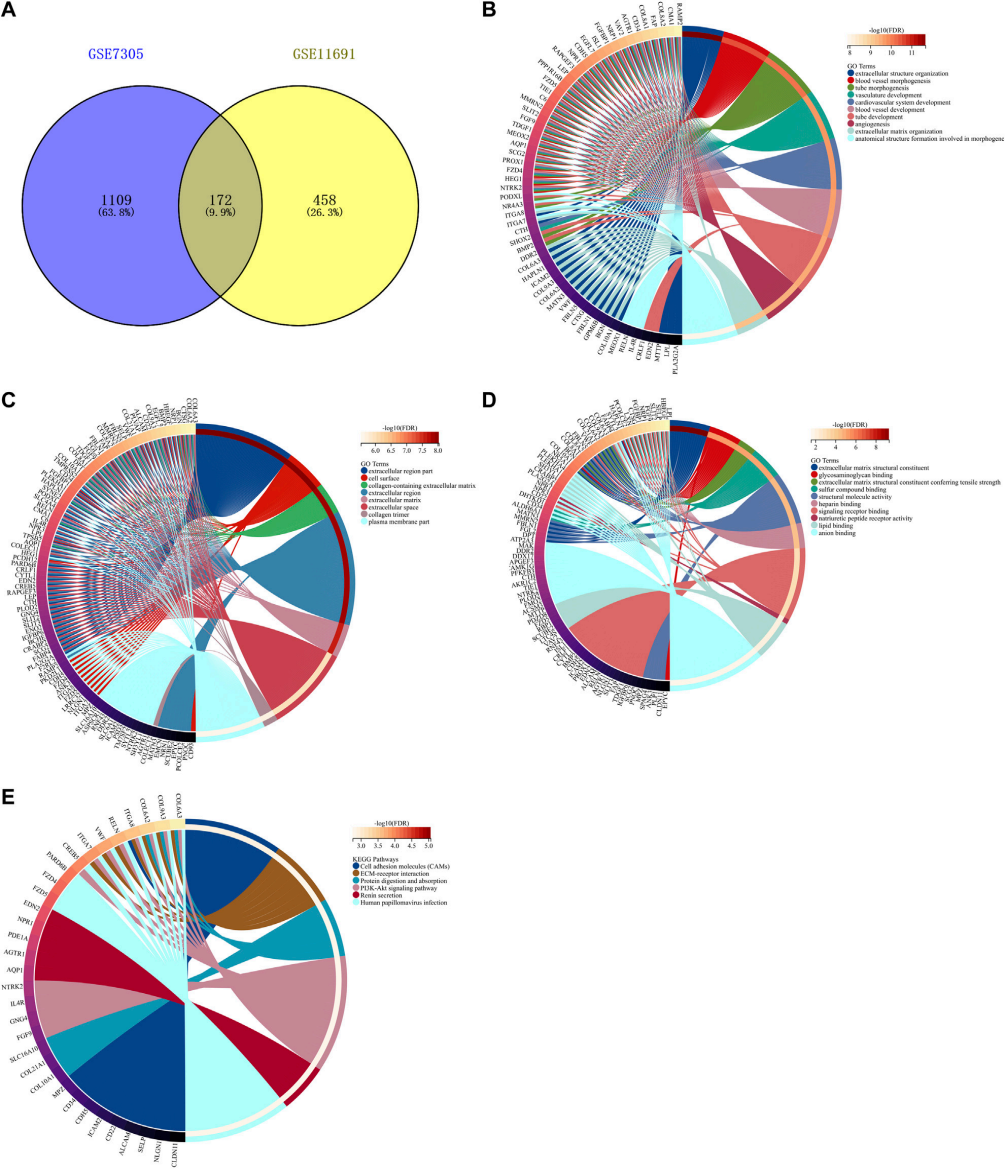
Identification of shared endometriosis-related genes in two datasets. **(A)** Venn diagram for shared endometriosis-related genes in the GSE7305 and GSE11691 datasets. **(B–E)** Biological processes, cellular components, molecular functions, and KEGG pathways enriched by shared endometriosis-related genes.

By taking the intersection of endometriosis-related genes in GSE7305 and GSE11691 datasets, we determined 172 shared endometriosis-related genes (Figure 3A, Supplementary Table S1). The shared endometriosis-related genes might mediate tube development, angiogenesis, and endometriosis-related pathways (PI3K-Akt pathway and extracellular matrix (ECM), etc.), demonstrating the crucial functions of the aforementioned genes in endometriosis (Figures 3B–E).

### Identification of four characteristic genes in endometriosis

**FIGURE 4:**
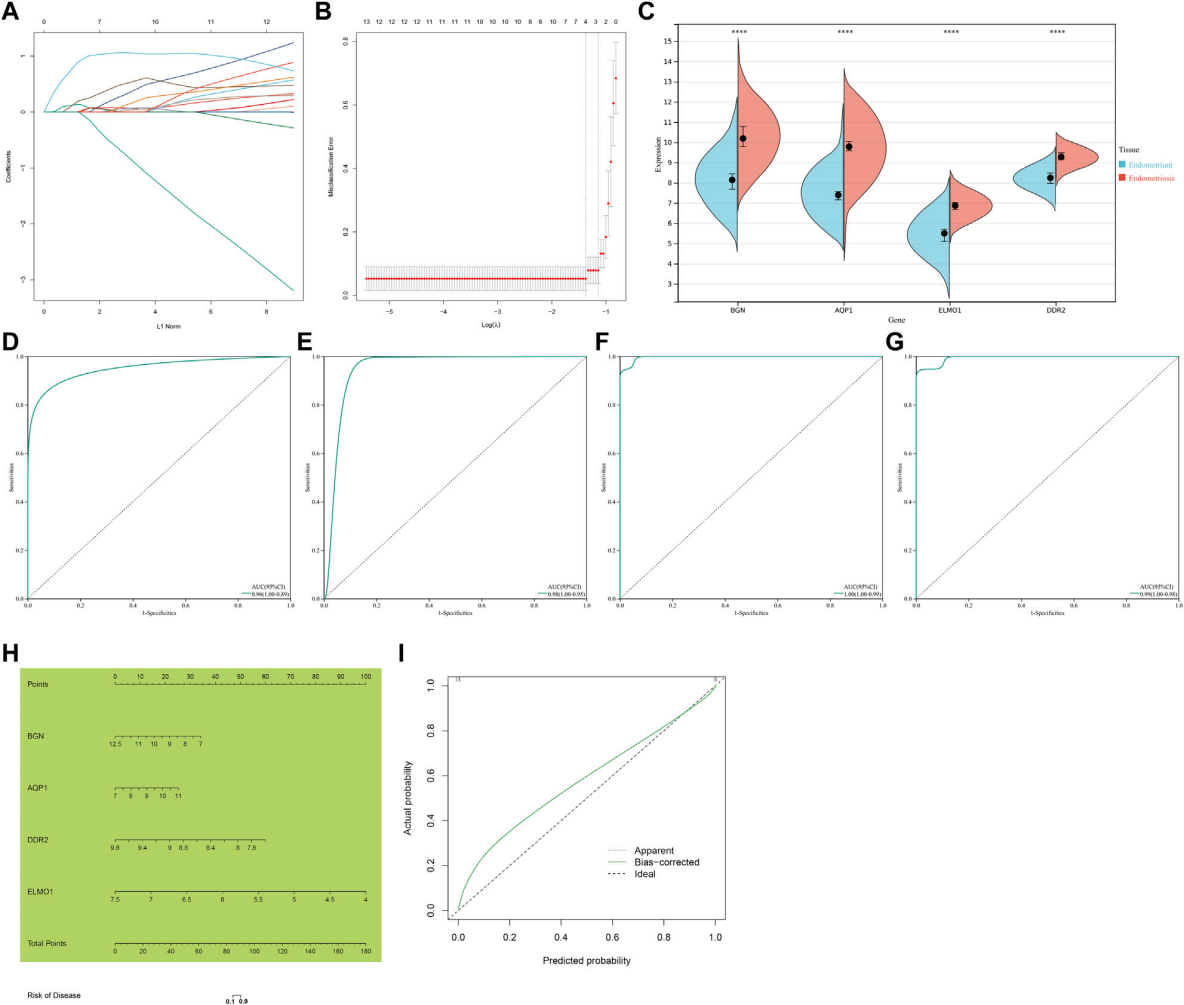
Identification of four characteristic genes and construction of a nomogram scoring system for endometriosis. **(A)** Lasso regression coefficients. Different colors represented different shared endometriosis-related genes. **(B)** Cross-verification for tuning the parameter selection. **(C)** Levels of four characteristic genes in endometriosis and normal endometrium tissues. ****p < 0.0001. **(D–G)** ROCs of four characteristic genes: AQP1, BGN, DDR2, and ELMO1. **(H)** Construction of a nomogram incorporating four characteristic genes. **(I)** Calibration plots showing the relationships between a nomogram and an ideal model in diagnosing endometriosis.

Through Lasso algorithm, four characteristic genes were determined among the shared endometriosis-related genes, containing BGN, AQP1, ELMO1, and DDR2 (Figures 4A,B). In the meta-dataset, their levels were significantly upregulated in endometriosis than normal endometrium tissues (Figure 4C). The AUCs (95%CI) of AQP1, BGN, DDR2, and ELMO1 were 0.96 (1.00–0.89), 0.98 (1.00–0.95), 1.00 (1.00–0.99), and 0.99 (1.00–0.98), respectively, demonstrating that each characteristic gene enabled to diagnose endometriosis accurately and sensitively (Figures 4D–G).

### Construction of a nomogram scoring system to diagnose endometriosis

Considering the convenience clinical utility, a nomogram incorporating all characteristic genes was constructed to diagnose endometriosis (Figure 4H). Calibration plots showed that the proposed nomogram exhibited the similar performance in comparison to an ideal model (Figure 4I), demonstrating the excellent predictive accuracy in endometriosis diagnosis.

### Verification of levels and diagnostic efficacy of characteristic genes in endometriosis

**FIGURE 5:**
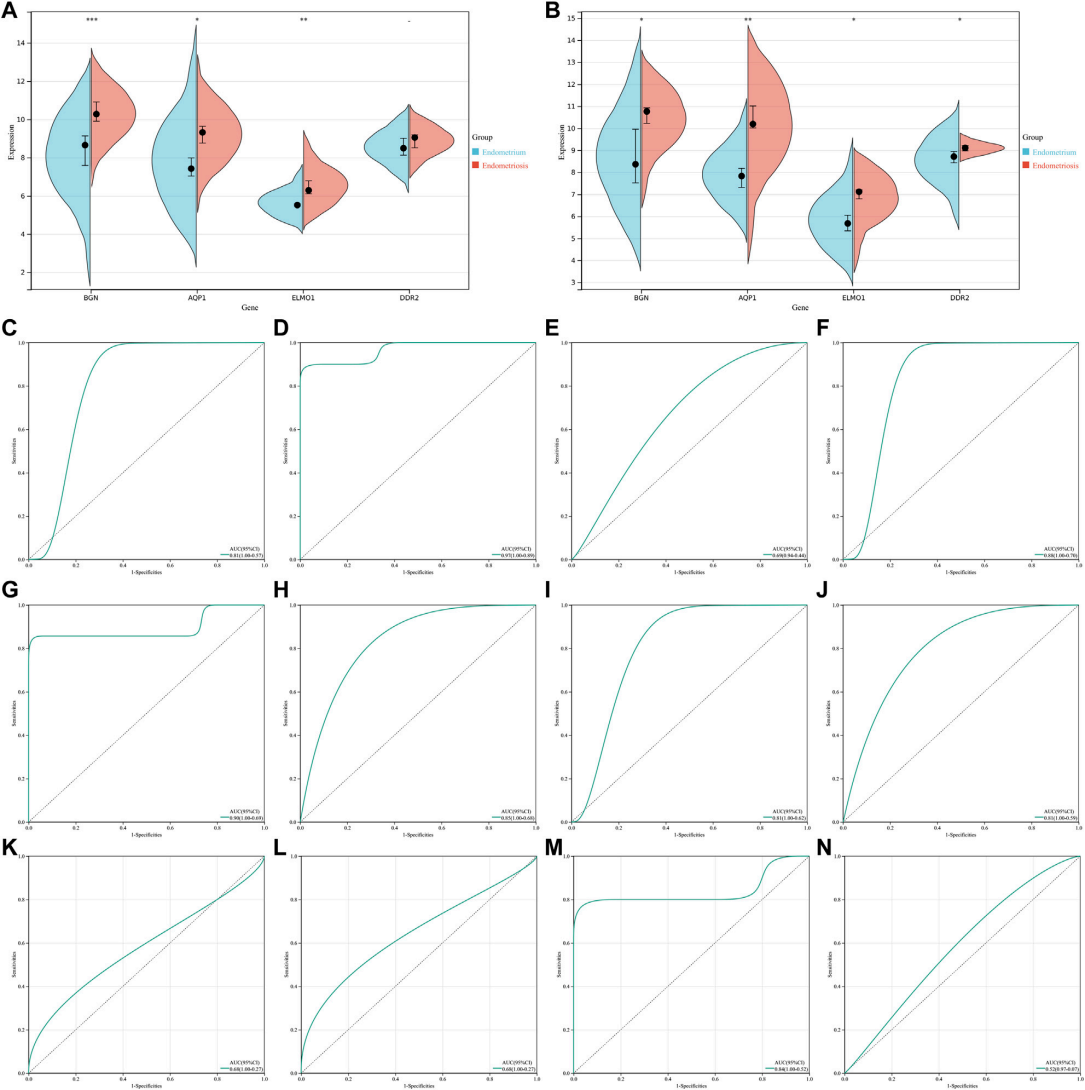
Verification of levels and diagnostic efficacy of characteristic genes in endometriosis. **(A,B)** Levels of four characteristic genes in endometriosis and normal endometrium tissues in GSE23339 and GSE25628 datasets. *p < 0.05; **p < 0.01; and ***p < 0.001. **(C–F)** ROCs of four characteristic genes: AQP1, BGN, DDR2, and ELMO1 in the GSE23339 dataset. **(G–J)** ROCs of four characteristic genes in the GSE25628 dataset. **(K–N)** ROCs of four characteristic genes in the GSE7846 external verification set.

The GSE23339 and GSE25628 datasets were employed for further verifying the levels and diagnostic efficacy of four characteristic genes in endometriosis. In the two datasets, higher levels of BGN, AQP1, ELMO1, and DDR2 were confirmed in endometriosis than normal endometrium tissues (Figures 5A,B). In the GSE23339 dataset, the AUCs (95%CI) of AQP1, BGN, DDR2, and ELMO1 were 0.81 (1.00–0.57), 0.97 (1.00–0.89), 0.69 (0.94–0.44), and 0.88 (1.00–0.70), respectively (Figures 5C–F). Meanwhile, in the GSE25628 dataset, the AUCs (95%CI) of BGN, AQP1, ELMO1, and DDR2 were 0.90 (1.00–0.69), 0.85 (1.00–0.68), 0.81 (1.00–0.62), and 0.81 (1.00–0.59), respectively (Figures 5G–J). Additionally, in the GSE7846 external verification set, the AUCs (95%CI) of the aforementioned characteristic genes were 0.68 (1.00–0.27), 0.68 (1.00–0.27), 0.84 (1.00–0.52), and 0.52 (0.97–0.07), respectively (Figures 5K–N). Following verifications, the four characteristic genes exhibited the well performance in diagnosing endometriosis.

### Signaling pathways involved in characteristic genes

**FIGURE 6:**
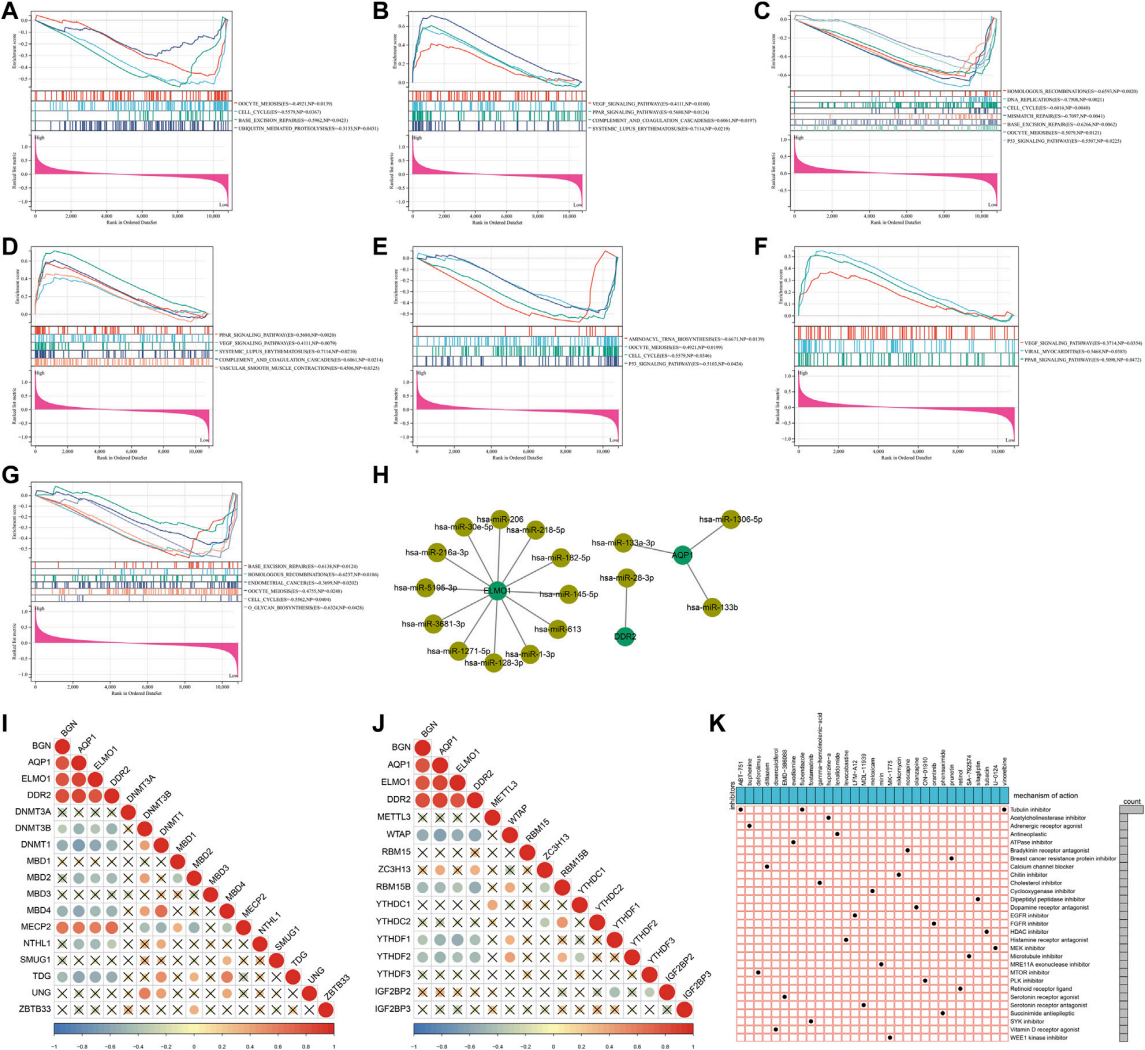
Signaling pathways and epigenetic and post-transcriptional modifications of characteristic genes and potential small compounds for endometriosis. **(A–H)** Signaling pathways significantly enriched by high or low levels of four characteristic genes: **(A,B)** AQP1, **(C)** BGN, **(D,E)** DDR2, and **(F,G)** ELMO1. **(H)** Regulatory network of miRNAs and characteristic genes. **(I,J)** Correlations of characteristic genes with **(I)** DNA and **(J)** m^6^A methylation regulators. **(K)** Shared mechanisms of action of small molecular inhibitors.

Through GSEA, signaling pathways involved in characteristic genes were analyzed. A low AQP1 level was linked to oocyte meiosis, cell cycle, base excision repair, and ubiquitin-mediated proteolysis (Figure 6A), and its high level was linked to VEGF signaling pathway, PPAR signaling pathway, complement and coagulation cascades, and systemic lupus erythematosus (Figure 6B). Homologous recombination, DNA replication, cell cycle, mismatch repair, base excision repair, oocyte meiosis, and p53 signaling pathway were correlated to the low BGN level (Figure 6C). Additionally, a high DDR2 level was associated with PPAR signaling pathway, VEGF signaling pathway, systemic lupus erythematosus, complement and coagulation cascades, and vascular smooth muscle contraction (Figure 6D), while its low expression was in relation to aminoacyl tRNA biosynthesis, oocyte meiosis, cell cycle, and p53 signaling pathway (Figure 6E). Also, VEGF signaling pathway, viral myocarditis, and PPAR signaling pathway were enriched in the high ELMO1 level (Figure 6F), while base excision repair, homologous recombination, endometrial cancer, oocyte meiosis, cell cycle, and O-glycan biosynthesis were enriched in the low ELMO1 level (Figure 6G). Altogether, characteristic genes might exert crucial roles in endometriosis through mediating the aforementioned signaling pathways.

### Post-transcriptional and epigenetic modifications of characteristic genes

At the post-transcriptional level, AQP1 was mainly regulated by hsa-miR-133a-3p, hsa-miR-133b, and hsa-miR-1306-5p; ELMO1 was modulated by hsa-miR-182-5p, hsa-miR-216a-3p, hsa-miR-218-5p, hsa-miR-1-3p, hsa-miR-128-3p, hsa-miR-145-5p, hsa-miR-206, hsa-miR-30e-5p, hsa-miR-613, hsa-miR-1271-5p, hsa-miR-3681-3p, and hsa-miR-5195-3p; and DDR2 was targeted by hsa-miR-28-3p (Figure 6H). The epigenetic modifications of characteristic genes were evaluated through calculating the associations of characteristic genes with DNA and m^6^A methylation regulators. As illustrated in Figure 6I, the characteristic genes were negatively linked to DNA methylation regulators DNMT3B, DNMT1, MBD2, MBD4, NTHL1, and TDG but positively linked to MECP2. Moreover, there were negative relationships of characteristic genes with m^6^A methylation regulators WTAP, RBM15B, YTHDF1, and YTHDF2 (Figure 6J). The aforementioned evidences demonstrated that the characteristic genes were modulated by post-transcriptional and epigenetic modifications in endometriosis.

### Prediction of potential drugs against endometriosis

In total, 413 genes with upregulation and 334 genes with downregulation were determined in endometriosis than normal endometrium (Supplementary Table S2). With scores ≤ −0.75, 51 drugs against endometriosis were determined (Table 1). Figure 6K depicted the shared mechanisms of action. For instance, ABT-751, flubendazole, and vinorelbine shared tubulin inhibitor.

**TABLE 1:** Potential drugs against endometriosis with scores ≤ −0.75.

Score	ID	Name	Description
−99.05	BRD-K15402119	Huperzine-a	Acetylcholinesterase inhibitor
−97.96	BRD-K35687265	ON-01910	PLK inhibitor
−97.45	BRD-K13927029	Retinol	Retinoid receptor ligand
−95.78	BRD-K05926469	Lenalidomide	Antineoplastic
−94.82	BRD-K20152659	Gamma-homolinolenic-acid	Cholesterol inhibitor
−94.28	BRD-K64785675	TG100-115	-666
−93.71	BRD-A69636825	Diltiazem	Calcium channel blocker
−93.57	BRD-A74771556	Nikkomycin	Chitin inhibitor
−93.51	BRD-A84174393	Meloxicam	Cyclooxygenase inhibitor
−92.66	BRD-K29733039	Deforolimus	MTOR inhibitor
−92.26	BRD-K22631935	Neurodazine	Neurogenesis of non-pluripotent C2C12 myoblast inducer
−92.21	BRD-A51393488	Noscapine	Bradykinin receptor antagonist
−90.8	BRD-K48427617	U-0124	MEK inhibitor
−89.23	BRD-K91696562	Orantinib	FGFR inhibitor
−89.16	BRD-A36267905	Buphenine	Adrenergic receptor agonist
−88.64	BRD-K91623615	ABT-751	Tubulin inhibitor
−88.14	BRD-A44551378	LFM-A12	EGFR inhibitor
−87.64	BRD-K86003836	Flubendazole	Tubulin inhibitor
−87.43	BRD-K98426715	Tubacin	HDAC inhibitor
−86.45	BRD-K47659338	EMD-386088	Serotonin receptor agonist
−86.25	BRD-K19416115	Sitagliptin	Dipeptidyl peptidase inhibitor
−85.19	BRD-K33453211	Levocabastine	Histamine receptor antagonist
−85.09	BRD-K14550461	Doxercalciferol	Vitamin D receptor agonist
−85.02	BRD-A68631409	Evodiamine	ATPase inhibitor
−84.49	BRD-A62057054	MDL-11939	Serotonin receptor antagonist
−83.64	BRD-A18043272	Phensuximide	Succinimide antiepileptic
−83.63	BRD-K57546357	Prunetin	Breast cancer resistance protein inhibitor
−82.59	BRD-M30523314	Vinorelbine	Tubulin inhibitor
−82.49	BRD-K55420858	Mirin	MRE11A exonuclease inhibitor
−81.82	BRD-K26997899	SA-792574	Microtubule inhibitor
−81.18	BRD-K18895904	Olanzapine	Dopamine receptor antagonist
−80.84	BRD-K54256913	MK-1775	WEE1 kinase inhibitor
−80.31	BRD-K20285085	Fostamatinib	SYK inhibitor
−79.76	BRD-K40213712	SAL-1	Adenosine receptor antagonist
−79.76	BRD-A00267231	Hemado	Adenosine receptor agonist
−79.76	BRD-K90382497	GW-843682X	PLK inhibitor
−79.71	BRD-K06878038	Deferiprone	Chelating agent
−79.6	BRD-A04756508	Norgestimate	Progesterone receptor agonist
−79.35	BRD-K29582115	Ziprasidone	Dopamine receptor antagonist
−78.81	BRD-A67438293	Treprostinil	Prostacyclin analog
−78.73	BRD-K99451608	Lopinavir	HIV protease inhibitor
−78.67	BRD-A74667430	Etodolac	Cyclooxygenase inhibitor
−78.22	BRD-K81376179	TCS-359	FLT3 inhibitor
−77.36	BRD-K67847053	Guanabenz	Adrenergic receptor agonist
−77.31	BRD-K27141178	SB-203186	Serotonin receptor antagonist
−77.14	BRD-A32836748	Leu-enkephalin	Opioid receptor agonist
−77.01	BRD-K53123955	Niridazole	Phosphofructokinase inhibitor
−76.48	BRD-K51318897	Fenbendazole	Tubulin inhibitor
−76.15	BRD-K11158509	Tyrphostin-B44	EGFR inhibitor
−75.99	BRD-K86465814	HO-013	PPAR receptor agonist
−75.51	BRD-K36324071	NF-449	Purinergic receptor antagonist

### Differences in immune features between endometriosis and normal endometrium

**FIGURE 7:**
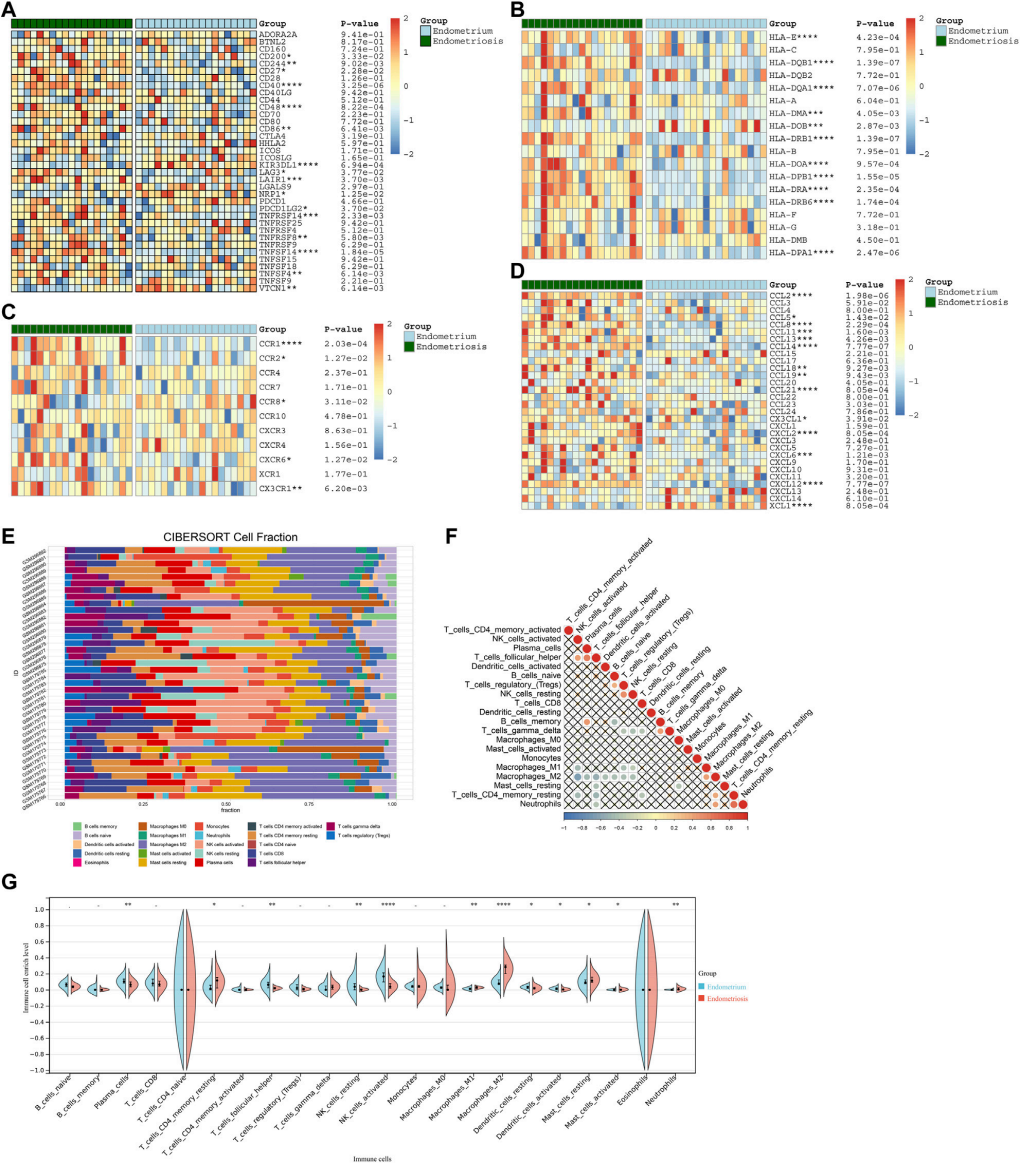
Differences in immune features between endometriosis and normal endometrium. **(A–D)** Heatmaps of the levels of **(A)** immune-checkpoints, **(B)** HLAs, **(C)** receptors, and **(D)** chemokines in endometriosis and normal endometrium tissues. **(E)** Fractions of 22 immune cell types across endometriosis and normal endometrium tissues. **(F)** Associations between immune cell types. **(G)** Abundance levels of immune cell types in endometriosis and normal endometrium tissues. *p < 0.05; **p < 0.01; ***p < 0.001; and ****p < 0.0001.

Immune features were evaluated in accordance with the levels of immune-checkpoints, HLAs, receptors, and chemokines as well as the abundance levels of immune cells. Most immune-checkpoints, HLAs, receptors, and chemokines displayed increased levels in endometriosis compared with normal endometrium tissues (Figures 7A–D). Utilizing the CIBERSORT algorithm, we estimated the relative proportions of 22 immune compositions across endometriosis and normal endometrium tissues, with macrophages occupying the highest proportion (Figure 7E). Figure 7F illustrated the tight interplay between these immune compositions, especially the macrophages were linked to most immune compositions. Moreover, most immune cells exhibited higher abundance levels in endometriosis than in normal endometrium tissues (Figure 7G).

### Associations of characteristic genes with immune features in endometriosis

**FIGURE 8:**
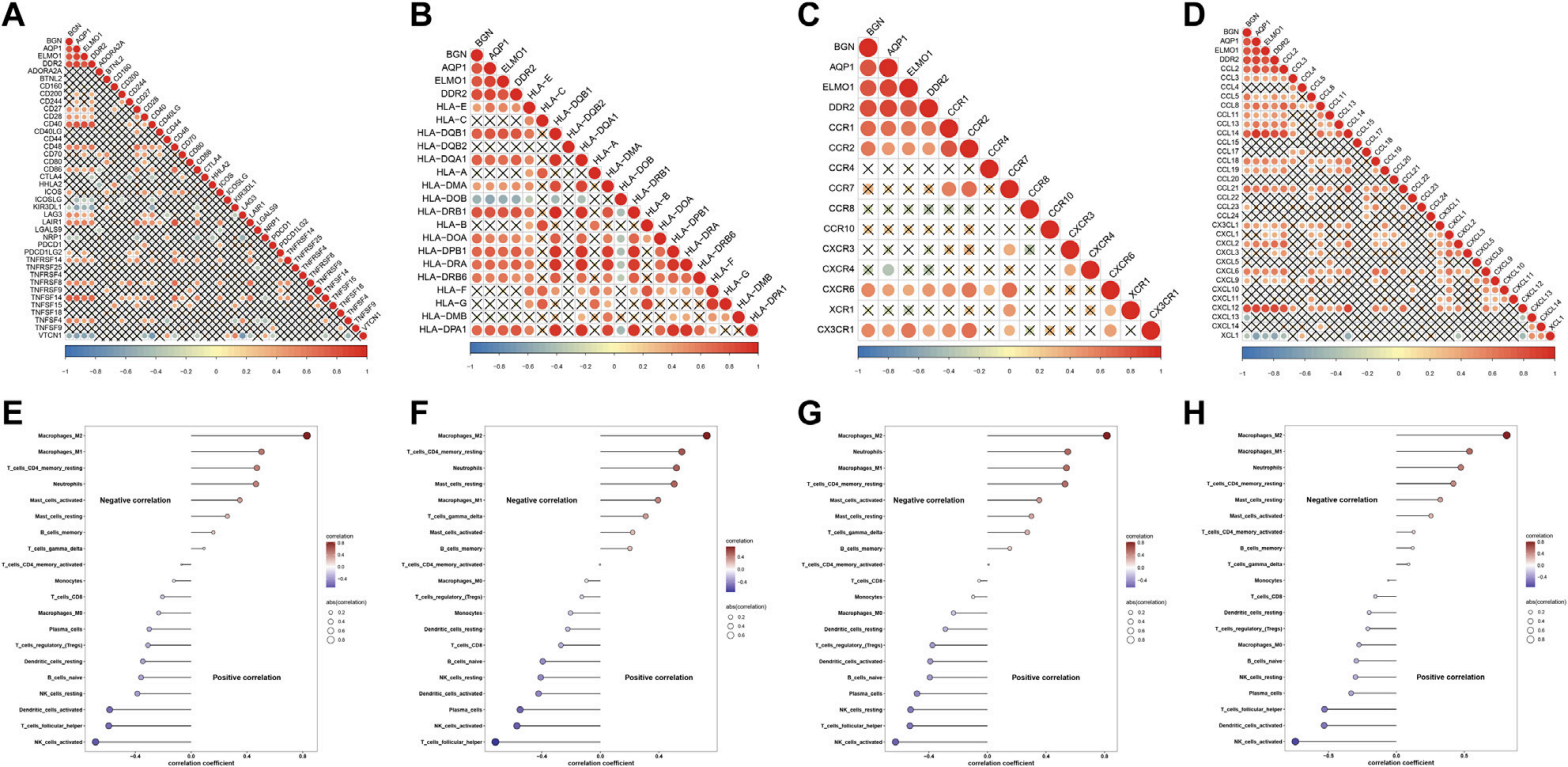
Associations of characteristic genes with immune features in endometriosis. **(A–D)** Heatmaps for the relationships of characteristic genes with **(A)** immune-checkpoints, **(B)** HLAs, **(C)** receptors, and **(D)** chemokines. **(E–H)** Correlations between characteristic genes: AQP1, BGN, DDR2, and ELMO1 and immune cell compositions.

Further analysis indicated that four characteristic genes: AQP1, BGN, DDR2, and ELMO1 exhibited positive correlations with most immune-checkpoints, HLAs, receptors, and chemokines (Figures 8A–D). Additionally, these characteristic genes were significantly linked with immune cell compositions, especially macrophages, NK cells activated, and follicular helper T cells (Figures 8E–H).

### Development of three subtypes for endometriosis

**FIGURE 9:**
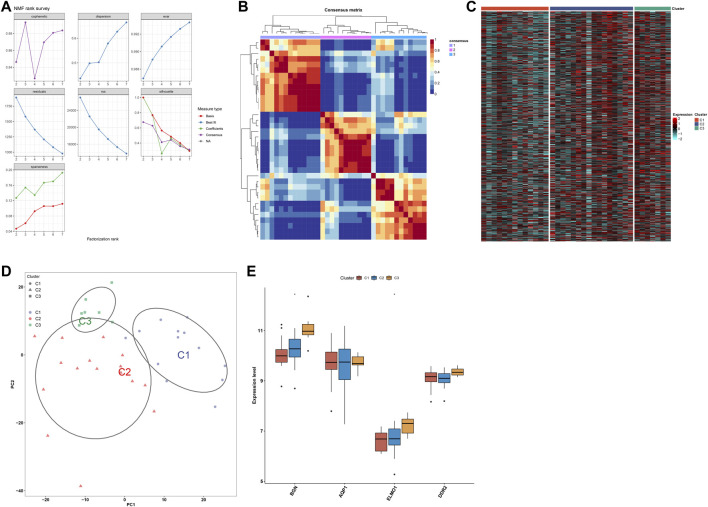
Development of three subtypes for endometriosis. **(A)** Cophenetic coefficients of the NMF clustering number from 2 to 7. **(B)** NMF matrix heatmap when k = 3. **(C)** Heatmaps of the expression patterns of endometriosis-related genes across three subtypes. **(D)** PCA of endometriosis-related genes. **(E)** Levels of four characteristic genes across three subtypes. *p < 0.05.

Utilizing the NMF algorithm, we classified endometriosis samples in the meta-dataset on the basis of endometriosis-related genes. Following cophenetic coefficients, k = 3 was determined as the optimal clustering number (Figure 9A). Figure 9B showed the NMF matrix when k = 3, containing 13 samples in C1, 16 samples in C2, and 7 samples in C3. The expression patterns of endometriosis-related genes were visualized in Figure 9C. PCA further complemented the distinction among three subtypes at transcription levels (Figure 9D). Additionally, four characteristic genes: BGN and ELMO1 levels were the highest in C3, followed by C2 and C1 (Figure 9E); no significant differences in AQP1 and DDR2 were detected among three subtypes.

### Differences in signaling pathways and immune features across three subtypes

**FIGURE 10:**
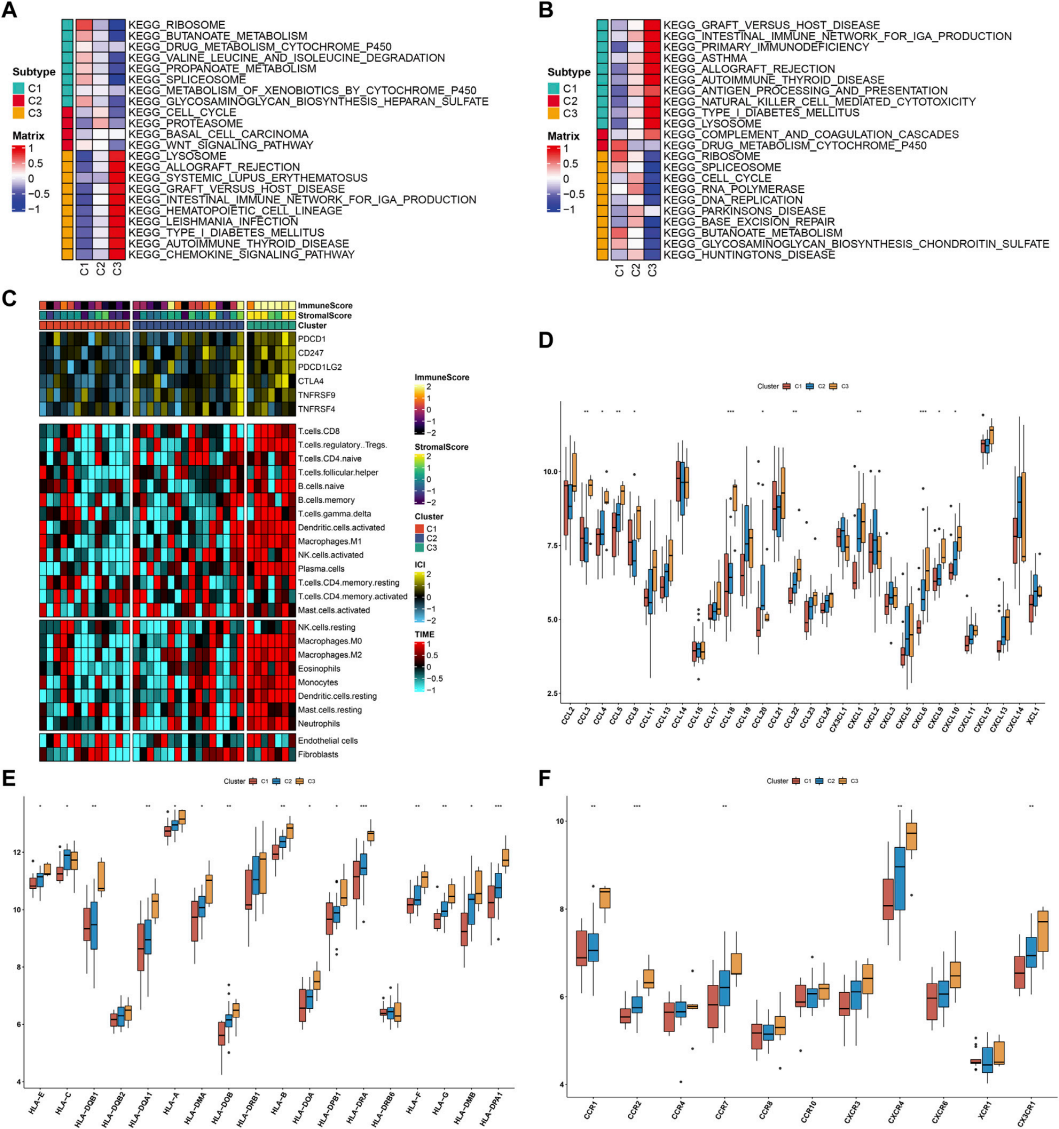
Differences in signaling pathways and immune features across three subtypes. **(A)** Upregulated pathways in each subtype. **(B)** Downregulated pathways in each subtype. **(C)** Heatmaps of immune-checkpoint levels, immune cell infiltrations, and immune and stromal scores across three subtypes. **(D–F)** Differences in **(D)** chemokines, **(E)** HLAs, and **(F)** receptors among three subtypes. *p < 0.05; **p < 0.01; and ***p < 0.001.

To uncover the signaling pathways underlying three subtypes, we evaluated the differences in signaling pathways among them. Upregulated pathways were as follows: ribosome, butanoate metabolism, drug metabolism cytochrome P450, valine, leucine, and isoleucine degradation, propanoate metabolism, spliceosome, metabolism of xenobiotics by cytochrome P450, and glycosaminoglycan biosynthesis heparan sulfate in C1 subtype; cell cycle, proteasome, basal cell carcinoma, and Wnt signaling pathway in C2 subtype; lysosome, allograft rejection, systemic lupus erythematosus, graft versus host disease, intestinal immune network for IgA production, hematopoietic cell lineage, leishmania infection, type I diabetes mellitus, autoimmune thyroid disease, and chemokine signaling pathway in C3 (Figure 10A). Downregulated pathways were as follows: graft versus host disease, intestinal immune network for IgA production, primary immunodeficiency, asthma, allograft rejection, autoimmune thyroid disease, natural killer cell-mediated cytotoxicity, type I diabetes mellitus, and lysosome in C1; complement and coagulation cascades, and drug metabolism cytochrome P450 in C2; ribosome, spliceosome, cell cycle, RNA polymerase, DNA replication, Parkinson’s disease, base excision repair, butanoate metabolism, glycosaminoglycan biosynthesis chondroitin sulfate, and Huntington’s disease in C3 (Figure 10B). C3 exhibited the highest of immune-checkpoint levels, immune cell infiltrations, and immune and stromal scores, followed by C2 and C1 (Figure 10C). Additionally, the levels of most chemokines, HLAs, and receptors were the highest in C3 along with C2 and C1 (Figures 10D–F). The aforementioned evidence demonstrated the differences in signaling pathways and immune features across three subtypes.

## Discussion

WGCNA is a system biology approach applied to describe gene association patterns between various samples, which can be applied to identify gene sets with highly coordinated changes, and to determine candidate organisms based on the interconnectivity of gene sets and the association between gene sets and phenotypes, thereby identifying marker genes or therapeutic targets. Through integrating GSE7305 and GSE11691 datasets, we determined 172 endometriosis-related genes utilizing WGCNA algorithm. Previously, endometriosis-related genes were determined utilizing the differential expression approach (Wang et al., 2021). Compared with only focusing on differentially expressed genes, WGCNA may use the information of thousands of genes with the greatest variations to identify gene sets of interest and implement significant association analysis with phenotypes (Wu et al., 2021). One is to make full use of the information, and the other is to convert the association between thousands of genes and phenotypes into associations between several gene sets and phenotypes, eliminating the problem of multiple hypothesis testing and correction. The endometriosis-related genes were linked to tube development, angiogenesis, and endometriosis-related pathways (PI3K-Akt pathway and ECM, etc.). Evidence proposes that angiogenesis, PI3K-Akt pathway, and ECM contribute to growth and progression of endometriotic cells within ectopic sites (Hung et al., 2021), demonstrating the crucial functions of the endometriosis-related genes in endometriosis.

Through the Lasso approach, we determined four characteristic genes among endometriosis-related genes, containing BGN, AQP1, ELMO1 and DDR2. All of them exhibited upregulated levels in endometriosis compared with normal endometrium tissues, which were modulated by post-transcriptional and epigenetic modifications. ROCs demonstrated that each characteristic gene enabled to diagnose endometriosis accurately and sensitively. Previously, upregulated BGN associated with estrogen metabolism and action in endometriosis was confirmed through immunohistochemical staining (Vouk et al., 2011). Suppression of AQP1 alleviates adhesion and angiogenesis of ectopic endometrial cells for murine endometriosis models via activation of the Wnt pathway (Shu et al., 2019). ELMO1 enables to increase the activity of extracellular matrix proteins as well as reduce cell adhesions to ECM (Shimazaki et al., 2006). Histological evidence demonstrates that endometriosis contributes to the increased incidence of ovarian cancer (Hermens et al., 2021). ELMO1 (Wang et al., 2014) and DDR2 (Jeong et al., 2021) have been demonstrated to mediate ovarian cancer progression. Altogether, the four characteristic genes we proposed might improve the early diagnosis as well as management of endometriosis cases.

In total, 51 drugs against endometriosis were determined. Among them, ABT-751, flubendazole, and vinorelbine shared tubulin inhibitor. The novel discovered small molecule compounds might exert a significant effect on the treatment of endometriosis. Endometriosis is a chronic neuroinflammatory disorder. Endometriosis exhibited increased levels of most immune-checkpoints, HLAs, receptors, and chemokines as well as enhanced infiltrations of most immune compositions compared with normal endometrium tissues (Peng et al., 2021). Consistent with the previous research, macrophages occupy the highest ratio among 22 immune cell components (Zhong et al., 2021). Recently, M2 macrophage-associated genes have been determined in endometriosis, reflecting the impact of M2 macrophages on the etiology of endometriosis (Cui et al., 2021). The four characteristic genes were positively correlated with most immune-checkpoints, HLAs, receptors, and chemokines as well as significantly linked with immune cell compositions, especially macrophages, NK cells activated, and follicular helper T cells, demonstrating that these characteristic genes might mediate immunological function during endometriosis progression. Determining the molecular subtypes of endometriosis is of importance for personalized treatment. With the NMF algorithm, we classified endometriosis as three subtypes that were linked to distinct signaling pathways and immune features.

The aforementioned findings might be beneficial for probing the pathogenesis of endometriosis as well as providing the foundation to determine novel biomarkers and subtypes for endometriosis. We believe that our findings will assist future research endeavors in the direction.

## Conclusion

Altogether, our research determined four characteristic genes (BGN, AQP1, ELMO1, and DDR2) with the favorable efficacy in diagnosing endometriosis. The characteristic genes were remarkably linked with immunological functions, and their aberrant levels were modulated by epigenetic and post-transcriptional modifications. Additionally, endometriosis was classified into three subtypes, with different mechanisms and immune features. The aforementioned findings might contribute to the early diagnosis and intervention in endometriosis.

## Data Availability

The datasets presented in this study can be found in online repositories. The names of the repository/repositories and accession number(s) can be found in the article/Supplementary Material.

## Abbreviations

WGCNA, weighted gene co-expression network analysis; NMF, non-negative matrix factorization; GEO, Gene Expression Omnibus; ROC, receiver operating characteristic; GSEA, gene set enrichment analysis; FDR, false discovery rate; Cmap, Connectivity Map; PCA, principal component analysis; AUC, area under the curve.
