# Supplementary figures and images for: Integrated bioinformatics analysis uncovers characteristic genes and molecular subtyping system for endometriosis
Source: Front Pharmacol. 2022 Aug 17;13:932526. doi: 10.3389/fphar.2022.932526 (PMC9428290; doi:10.3389/fphar.2022.932526)

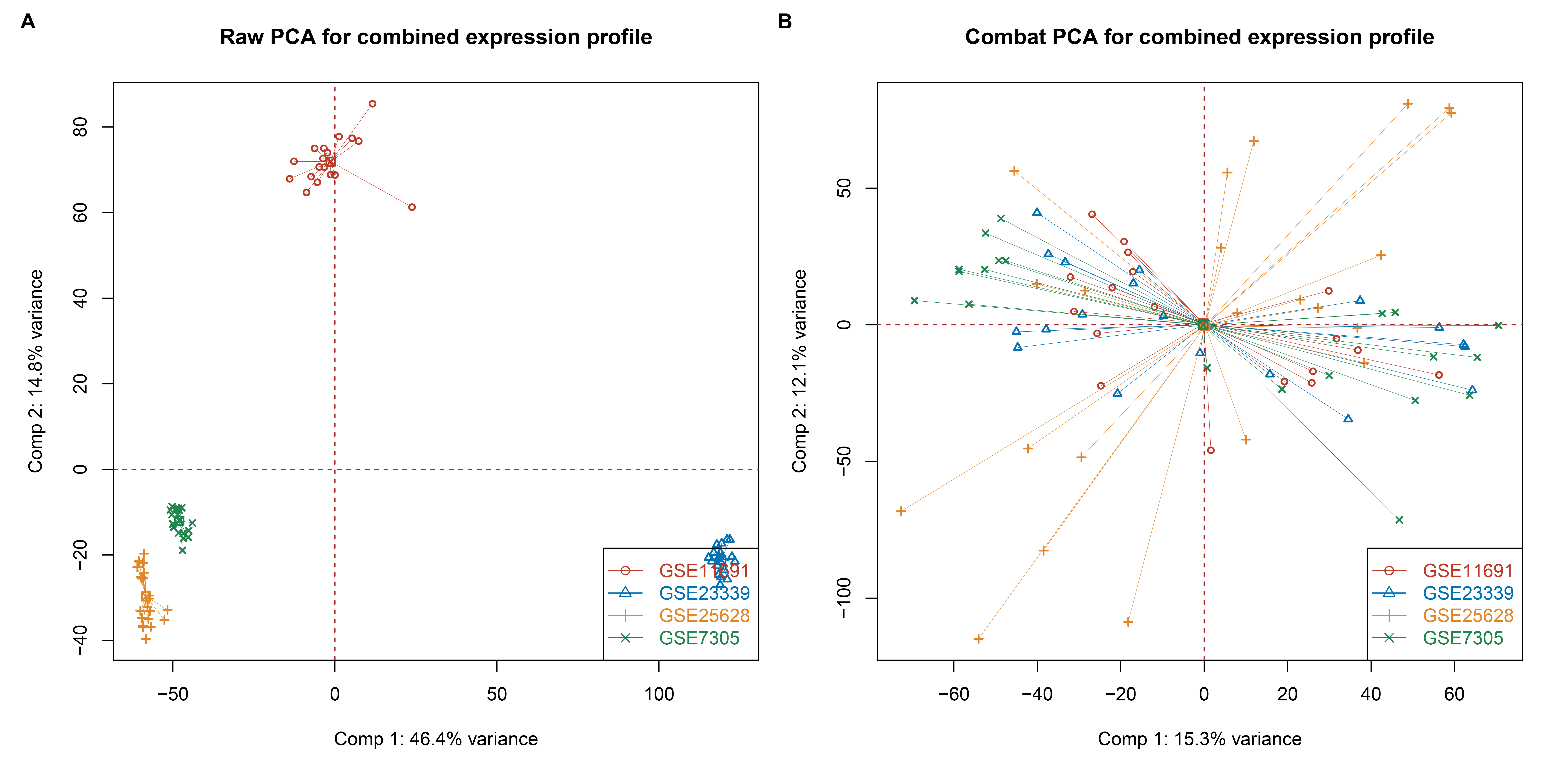

Supplement: Supplementary file 2 [file Image1.TIF]
